# Blood transcriptome responses to PFOA and GenX treatment in the marsupial biomedical model *Monodelphis domestica*

**DOI:** 10.3389/fgene.2023.1073461

**Published:** 2023-02-15

**Authors:** Wenqi Cao, Katharine Horzmann, Bettina Schemera, Myra Petrofski, Trisha Kendall, Jennifer Spooner, Patricia E. Rynders, John L. VandeBerg, Xu Wang

**Affiliations:** ^1^ Department of Pathobiology, College of Veterinary Medicine, Auburn University, Auburn, AL, United States; ^2^ Alabama Agricultural Experiment Station, Auburn University Center for Advanced Science, Innovation, and Commerce, Auburn, AL, United States; ^3^ Division of Laboratory Animal Health, College of Veterinary Medicine, Auburn University, Auburn, AL, United States; ^4^ Department of Human Genetics, School of Medicine, South Texas Diabetes and Obesity Institute, The University of Texas Rio Grande Valley, Brownsville, TX, United States; ^5^ HudsonAlpha Institute for Biotechnology, Huntsville, AL, United States

**Keywords:** per-and polyfluoroalkyl substances (PFAS), laboratory opossum, long-chain fatty acid transport, inflammatory response, developmental processes, toxicology

## Abstract

**Introduction:** Perfluoroalkyl and poly-fluoroalkyl substances (PFASs) are widely used in industrial and consumer products. Due to their environmental persistence and bioaccumulation, PFASs can be found in the blood of humans and wild animals all over the world. Various fluorinated alternatives such as GenX have been developed to replace the long-chain PFASs, but there is limited information about their potential toxicity.

**Methods:**The current study developed blood culture protocols to assess the response to toxic compounds in the marsupial, *Monodelphis domestica*. After whole-blood culture conditions were tested and optimized, changes in gene expression in response to PFOA and GenX treatment were assessed.

**Results:** More than 10,000 genes were expressed in the blood transcriptomes with and without treatment. Both PFOA and GenX treatment led to significant changes in the whole blood culture transcriptomes. A total of 578 and 148 differentially expressed genes (DEGs) were detected in the PFOA and GenX treatment groups, 32 of which overlapped. Pathway enrichment analysis revealed that DEGs involved in developmental processes were upregulated after PFOA exposure, while those enriched for metabolic and immune system processes were downregulated. GenX exposure upregulated genes associated with fatty acid transport pathways and inflammatory processes, which is consistent with previous studies using rodent models.

**Discussion:** To our knowledge, this study is the first to investigate the effect of PFASs in a marsupial model. The findings provide supportive evidence for significant transcriptomic alterations, suggesting that this mammalian model may provide a mechanism for exploring the potential toxicity of PFOA and GenX.

## Introduction

Perfluoroalkyl and poly-fluoroalkyl substances (PFASs) are manufactured chemicals that have been widely used in the manufacture of cosmetics, firefighting foams, food contact materials, textiles, and other industrial and consumer products (Giesy and Kannan, 2002). There are thousands of different PFASs with fluorinated carbon chains of varying lengths that contain different functional groups (Buck et al., 2011). The high level of PFAS persistence, bioaccumulation, and use has contributed to their ubiquitous presence in the environment, especially in drinking water. Most PFASs are non-degradable or break down very slowly, allowing them to build up in organisms and the environment over time. Perfluorooctanoic acid (PFOA) and perfluorooctanesulfonic acid (PFOS) are two of the most widely used long-chain PFASs.

Human studies have found that some long-chain PFASs such as PFOS and PFOA tend to accumulate in the kidney, liver, and blood (Verreault et al., 2005), where they bind to serum albumin (Jones et al., 2003). PFAS concentration is determined using whole blood, plasma, or serum samples. Measurements in whole blood samples are approximately one-half of those detected in human plasma or serum (Ehresman et al., 2007). While PFOA has an average half-life of 3.8 years in serum, the half-life of PFOS is even longer (Olsen et al., 2007). Epidemiologic and animal-based research studies have linked PFAS exposure with cancer, impaired development, and detrimental effects on the immune system and reproductive function (Suh et al., 2011; McDonough et al., 2020; Grønnestad et al., 2021; Imir et al., 2021; Roth et al., 2021; Tarapore and Ouyang, 2021). Metabolic pathways, including fatty acid metabolism, are also affected (Sunderland et al., 2019).

Long-chain PFASs are recognized as environmental contaminants of serious concern due to their PBT characteristics (P: high persistence, B: bioaccumulation potential, T: toxicity). As a result, several long-chain PFASs, including PFOA and PFOS, have been gradually phased out in the US and Europe since 2000. They are replaced by various fluorinated alternatives, including the shorter-chain homologs of long-chain perfluoroalkyl acids and their precursors, as well as functionalized perfluoropolyethers (PFPEs) (Wang et al., 2013). GenX is the trade name for the ammonium salt of hexafluoropropylene oxide dimer acid (NH4-HFPO-DA), one of the most widely used fluorinated alternatives. This substance has been detected worldwide, including in the air and drinking water (Strynar et al., 2015; Sun et al., 2016; Gebbink et al., 2017), and persists for long periods in the environment (Wang et al., 2015). A recent study using electrooxidation found that GenX has a significantly slower rate of degradation than PFOA in water (Yang et al., 2022). Limited studies using rats and mice have shown that GenX has a shorter serum elimination half-life (Wang et al., 2013; Beekman et al., 2016). Recent animal experiments have found that GenX is also associated with reported adverse effects on neonatal development, lipid metabolism, and immune system function (Blake et al., 2020; Wen et al., 2020; Conley et al., 2021). In rat and human thyroid cell lines, GenX is shown to be more toxic than PFOA (Zhang et al., 2021). Acute toxicity, genotoxicity, and developmental toxicity have also been reported (Gordon, 2011; Gomis et al., 2018). The current study sought to complement rodent models by assessing the effects of PFAS exposure on a novel laboratory animal model, *Monodelphis domestica*.

The gray short-tailed opossum, *Monodelphis domestica*, is the best marsupial model for biomedical research (Fadem et al., 1982). This animal has been studied in a research laboratory environment for more than 40 years, has a well-assembled and well-annotated reference genome (Mikkelsen et al., 2007), and includes two validated inbred strains that enable control of its genetic background (Xiong et al., 2022). Marsupials (metatherian mammals) diverged from placentals (eutherian mammals) approximately 173–190 million years ago (Woodburne et al., 2003; van Rheede et al., 2006; Luo et al., 2011). The fundamental physiological and molecular processes of the opossum are conserved compared to eutherian mammals, making them an excellent alternative mammal model in which to identify highly conserved key genes and important functional pathways (Samollow, 2008). The current study performed RNA sequencing experiments in *Monodelphis domestica* to identify blood transcriptome changes that occurred in response to PFAS treatment. The effects of PFOA, one of the most widely used and most common long-chain PFAS in drinking water (Post et al., 2012), and GenX, one of the most popular PFOA/PFOS alternatives used in the industry, were assessed. The aim was to establish a blood cell culture system in the laboratory opossum to determine the effects of PFOA and GenX exposure on global gene expression, and to lay the foundation for further animal study of the impact of environmental pollutants on early embryonic development.

## Materials and methods

### Animal maintenance

The *Monodelphis domestica* used in this study was the LSD strain from a breeding colony established at Auburn University, with founder animals obtained from Dr. John VandeBerg’s laboratory at the University of Texas Rio Grande Valley. The animal maintenance, breeding, and operations followed the standard operating procedures (SOP) approved by the Auburn University Institutional Animal Care and Use Committee (AU-IACUC, approval numbers 2018-3,277 and 2021-3,883). The animals were individually caged under laboratory conditions at 23.5°C–26.5°C and 55%–60% humidity. A 14 h/10 h light/dark fluorescent lighting cycle was used. Dedicated commercial food and local municipal water were used as the daily supply. Daily care and veterinary services were provided by the Division of Laboratory Animal Health staff. A total of ten animals were used in the two experiments (Supplementary Table S1).

### Blood sample collection and whole blood culture

**FIGURE 1:**
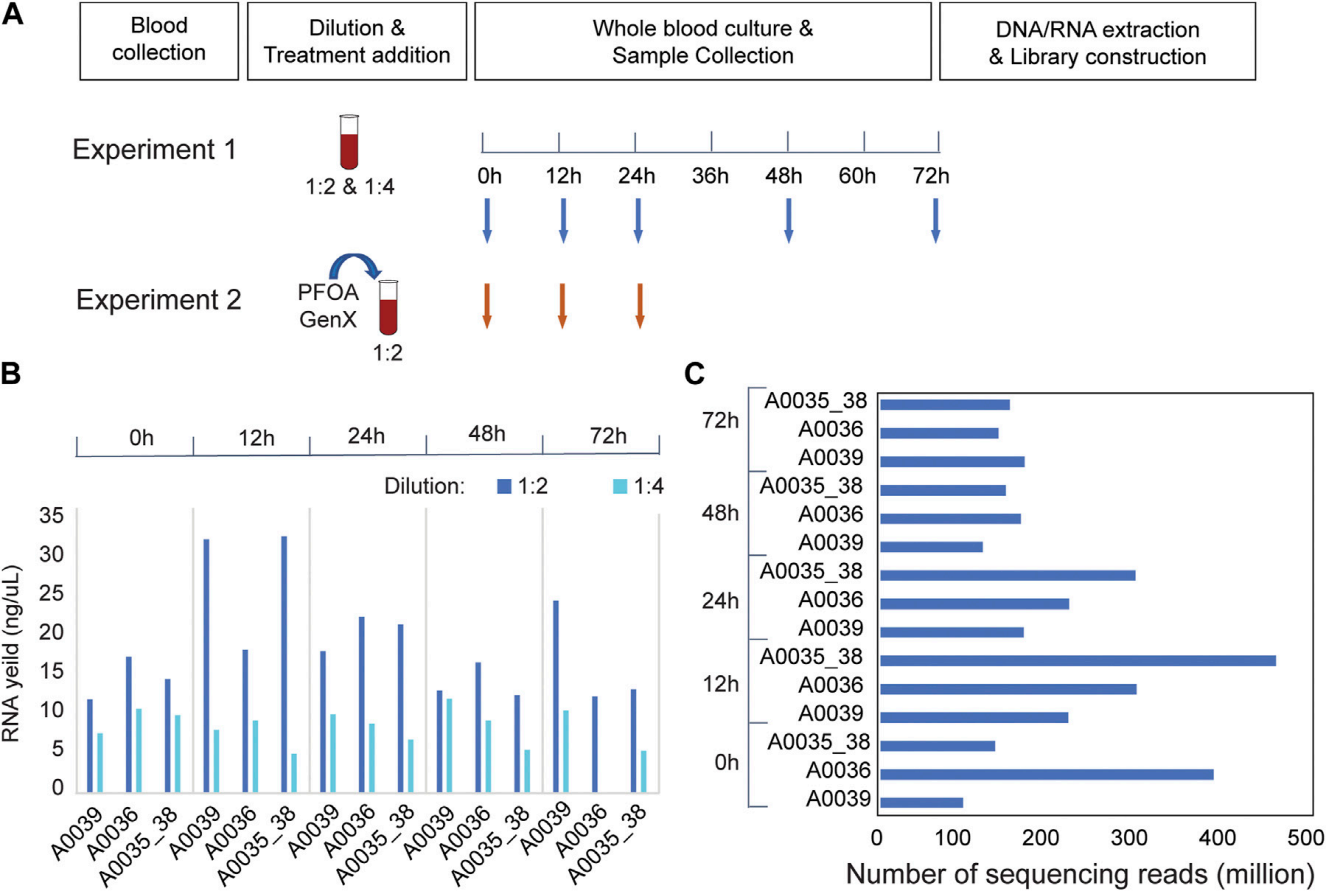
RNA-seq experiments in *Monodelphis domestica* whole blood culture samples without chemical disturbance. **(A)** Design of pilot experiments for protocol development (Experiment 1) and toxicity (Experiment 2). **(B)** RNA yield of *Monodelphis domestica* whole blood culture samples with different dilution factors and culture times. **(C)** RNA sequencing yield in Experiment 1.

Blood samples were obtained from anesthetized animals by cardiac puncture according to the IACUC-approved procedure for *Monodelphis domestica*. In brief, 1 mL sterile syringes (U-100, Becton, Dickinson and Company, NJ) were used for the blood draw, and samples were temporarily stored in a 2 mL vacutainer with lithium heparin (Becton, Dickinson and Company, NJ). In pilot Experiment 1, three biological replicates were included (A0036, A0039, and A0035_38). The blood samples from A0035 and A0038 were pooled to achieve a sufficient volume for culture experiments. The samples were diluted 1:2 and 1:4 with sterile RPMI-1640 culture medium complemented with 2 mM glutamine and antibiotics (100 U/mL penicillin and 100 μg/mL streptomycin), transferred to sterile 12-well culture plates with lids (USA Scientific, FL), and incubated at 37°C with 5% CO2. In Experiment 2, blood samples collected from two males (A0093 and A0094) were pooled to achieve a sufficient volume. Female blood samples were excluded from the analysis because insufficient amounts of blood were collected. The blood samples were diluted 1:2 with the same culture medium and cultured under the same conditions. Different exposure times were selected. In Experiment 1, 200 μl of the mixture was extracted at 0, 12, 24, 48, and 72 h after incubation (Figure 1A). In Experiment 2, 400 μl of blood culture was extracted at 0, 12, and 24 h after incubation. Three replicates were collected under each treatment condition at each time point. The samples were stored at −80°C before the extraction of DNA/RNA.

### PFOA and GenX treatments

PFOA and GenX were purchased from Sigma-Aldrich (Chemical Abstracts Service Registry numbers PFOA:335-67-1; GenX: 62037-80-3). Stock solutions were freshly prepared by dissolving the chemicals in molecular-grade dimethyl sulfoxide (DMSO) (>99.9%). The DMSO concentration was calculated and adjusted to achieve a final 0.1% volume percent (v/v) in all treatment and control groups. The desired concentrations in the final blood culture were achieved by adjusting the PFOA and GenX stock solutions. Two concentrations of each chemical were used in Experiment 2 (PFOA: 400 μM and 800 μM; GenX: 600 μM and 1,200 μM). DMSO of the same volume was added to the control group without treatment.

### DNA/RNA extraction, RNA-seq library preparation, and Illumina sequencing

DNA/RNA extractions were conducted using Zymo Quick-DNA/RNA Miniprep Plus Kit (Zymo Research, CA) according to the manufacturer’s instructions. DNA/RNA quantity was measured using the Qubit Fluorometer 3.0 (Thermo Fisher Scientific, MA) with the Qubit RNA BR Assay Kit. RNA samples with >100 ng yield were selected for RNA-seq library preparation. RNA sequencing libraries were constructed using the NEBNext Ultra II Directional RNA Library Prep Kit for Illumina (New England Biolabs, MA) and NEBNext Poly(A) mRNA Magnetic Isolation Module (New England Biolabs, MA). The final concentrations and size distribution of the libraries were checked using the Qubit and HT DNA NGS 3K Assay on a LabChip GX Touch HT machine (Perkin Elmer, MA) (Supplementary Table S2). Libraries were sent for sequencing on an Illumina NovaSeq 6000 sequencing machine to generate 150-bp paired-end reads.

### Analysis of the RNA-seq data

RNA-seq read quality was checked using FastQC v11.5 (Andrews, 2010). Trimmomatic v0.36 was used to remove Illumina adapter sequences and low-quality bases (Bolger et al., 2014). After trimming, 98.2% of the reads survived the quality control filtering in Experiment 1 and 96.4% in Experiment 2. The high-quality reads were mapped to the *Monodelphis domestica* reference genome assembly (momDom5, GCA_000002295.1) by Tophat (version 2.1.1) and STAR (version 2.7.5) (Trapnell et al., 2009; Langmead and Salzberg, 2012; Dobin et al., 2013).

### Identification of differentially expressed genes (DEGs)

A total of 10 transcriptomes of the same sex (male) were used for analysis. Two control untreated samples, two PFOA-treated, and two GenX-treated samples were included 12 h after treatment. Two control untreated samples and two PFOA-treated samples were included 24 h after treatment. At 24 h, the GenX-treatment may suffer from a degradation issue (Liberatore et al., 2020), and was excluded from the analysis. Gene counts were summarized using BEDTools version 2.30.0 (Trapnell et al., 2009; Quinlan and Hall, 2010). Normalization of raw counts and identification of DEGs were performed using the TMM method associated with the edgeR package in R (version 4.1.2) (Robinson et al., 2010). Adjusted *p*-values were computed using the Benjamini–Hochberg method, with a False Discovery Rate (FDR) of <0.05. The cut-off for detecting significant DEGs was |log2(fold change)| >1 and FDR <0.05. Individual gene expression levels were quantified by Read Per Kilobase of transcript per Million mapped reads (RPKM). MA and volcano plots were generated in R.

### Functional pathway analysis of the DEGs

Functional enrichment analysis was performed using Metascape with default parameters (Zhou et al., 2019), for upregulated and downregulated DEGs identified in the PFOA and GenX groups, respectively. Gene Ontology (GO) term analyses focusing on biological processes were performed at an adjusted *p*-value cut-off of 0.01.

### Quantitative reverse-transcription PCR (qRT-PCR) validation of selected DEGs

Ten DEGs were selected for qRT-PCR validation, including three downregulated DEGs (*Itgax*, *Alox15*, and *Plin2*) and seven upregulated genes (*Ptn, Aldh1a5*, *Wdr47*, *Cd36*, *Cd38*, *Fabp5*, and *Itgb3*). The housekeeping gene, *Gapdh,* was selected as a reference gene for normalization based on relatively equal expression levels (as measured by RPKM) in the control and treated samples. Reverse transcription was performed using the LunaScript RT SuperMix Kit (New England Biolabs, MA). The input of the total RNA in each sample was 20 ng. A 20 μl reaction system was incubated for 2 min at 25°C, followed by 10 min at 55°C and 1 min at 95°C. QPCR primers were designed using Oligo 7.0 (Molecular Biology Insights Inc., CO) and synthesized at Eurofins Genomics (Supplementary Table S3). The UCSC In-Silico PCR tool was used to check the primer specificity in the *Monodelphis domestica* genome and transcriptome. QRT-PCR assays were performed with SYBR Green using the Luna Universal qPCR Master Mix kit (New England BioLabs, MA) in 96-well plates on a BioRad CFX Opus 96 thermocycler (Bio-Rad Laboratories, CA). The initial denaturing step was set at 95°C for 30 s, followed by 39 cycles of denaturation for 10 s at 95°C and extension for 30 s at primer-specific extension temperature. The melting curve was generated by heating from 65°C to 95°C in 0.5°C increments, with a 5 s dwell time. Two technical replicates were performed for each selected gene, and a t-test was used to measure differences in gene expression.

## Results

### A blood culture model of PFAS exposure in the laboratory opossum, *Monodelphis domestica*

To optimize the experimental conditions for blood culture in the laboratory opossum, a pilot experiment (Experiment 1) was performed using two different treatment dilutions in culture media (1:2 and 1:4). The blood mixtures were cultured for 72 h, and 200 μL samples were collected at 0, 12, 24, 36, and 72 h after culture (Figure 1A). At a 1:2 dilution, the total RNA yield was higher than 1:4, and the 12 and 24 h cultures had better RNA yield and quality than the 48 or 72 h cultures (Figure 1B). In the RNA-seq experiments, an average of 178.5 million mapped reads were obtained per sample, and a total of 11,349 expressed genes were detected in the blood transcriptome (Figure 1C). Transcriptome-wide expression correlations were high for the first 36 h after culture (Spearman’s rank correlation coefficient ρ = 0.9; Supplementary Figure S1A) after which they decreased significantly (ρ < 0.8; Supplementary Figure S1A). There were strong positive correlations between the two biological replicates (ρ > 0.95; Supplementary Figure S1B) at 12, 24, and 48 h after treatment that decreased at 72 h (Supplementary Figure S1B). Thus, up to 36 h of culture, extremely high correlations were observed between replicates and sufficient correlations were observed between time points.

### Blood transcriptome analysis of *Monodelphis domestica* whole blood culture samples after PFOA/GenX treatment

Based on the results of pilot Experiment 1, PFOA and GenX treatments (Experiment 2) were performed in blood culture with samples collected at 12 and 24 h (Figure 1B). Two different concentrations of PFOA (400 μM and 800 μM) and GenX (600 μM and 1,200 μM) were used. Two biological replicates were included for RNA sequencing library preparation of the control, PFOA, and GenX treatment at 12 and 24 h, and the average insert size for the final libraries was 302 bp. Samples from the GenX group were excluded from the RNA-seq analysis at 24 h due to technical issues (see Methods). On average, 18.3 million mapped 150-bp reads were obtained per sample. A total of 10,940 and 11,470 expressed genes were detected in the PFOA and GenX treatment groups, respectively, which was similar to Experiment 1.

### Identification of DEGs following exposure to PFOA and GenX

**FIGURE 2:**
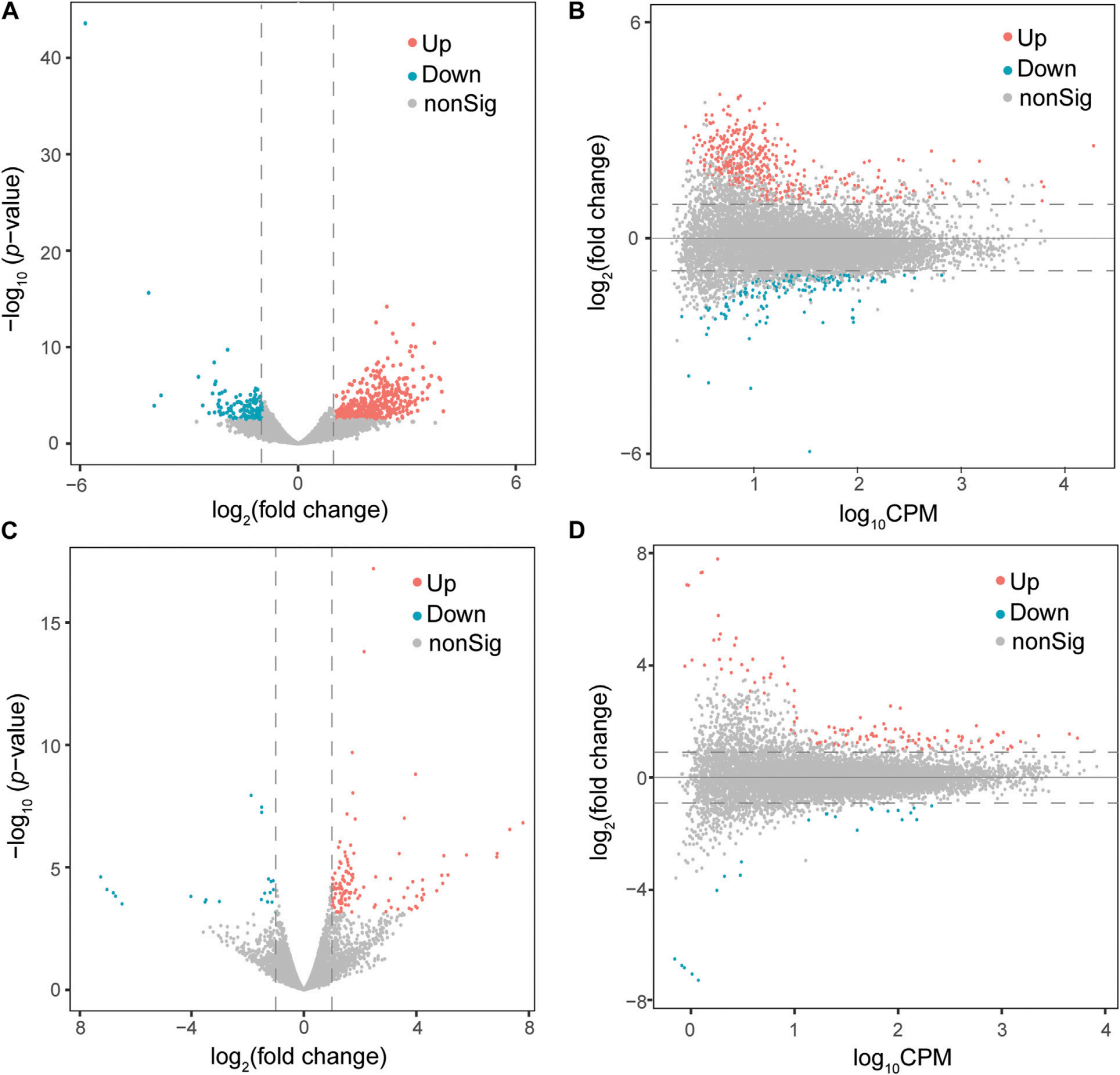
Differential gene expression analysis of male *Monodelphis domestica* blood culture samples from the PFOA/GenX treatment and control groups. **(A, C)** Volcano plot demonstrating differentially expressed genes (DEGs) in the PFOA **(A)** and GenX **(C)** treatment groups compared to the control group. Red dots represent significantly upregulated genes and blue dots represent significantly downregulated genes (FDR <0.05 and |log_2_FoldChange| >1). Grey dots represent non-significant genes. **(B, D)** MA plot demonstrating the DEGs in the PFOA **(B)** and GenX **(D)** treatment groups compared to the control group. The *x*-axis is log_10_CPM (log_10_ Counts per Million). The *y*-axis is log_2_ (Fold Change). The horizontal line indicates |log_2_FoldChange| = 1. Red dots indicate genes with significantly increased expression in the PFOA treatment group compared to the control group, and blue dots indicate genes with significantly decreased expression (FDR <0.05 and |log_2_FoldChange| >1).

**FIGURE 3:**
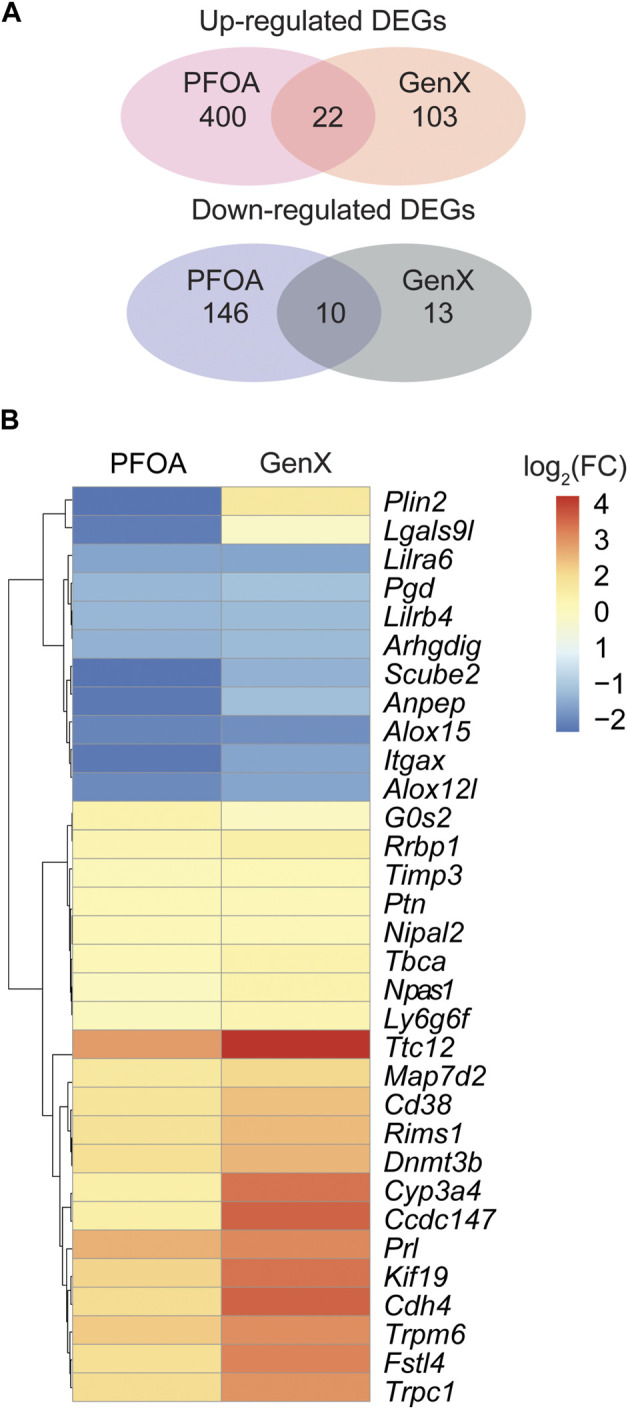
Differentially expressed genes in the PFOA and GenX-treated groups. **(A)** Venn diagram showing the overlap of upregulated and downregulated DEGs among the two treatment groups. **(B)** Hierarchical clustering of 32 overlapped DEGs identified between the PFOA and GenX treatment groups. Red represents upregulation, and blue indicates downregulation.

**FIGURE 4:**
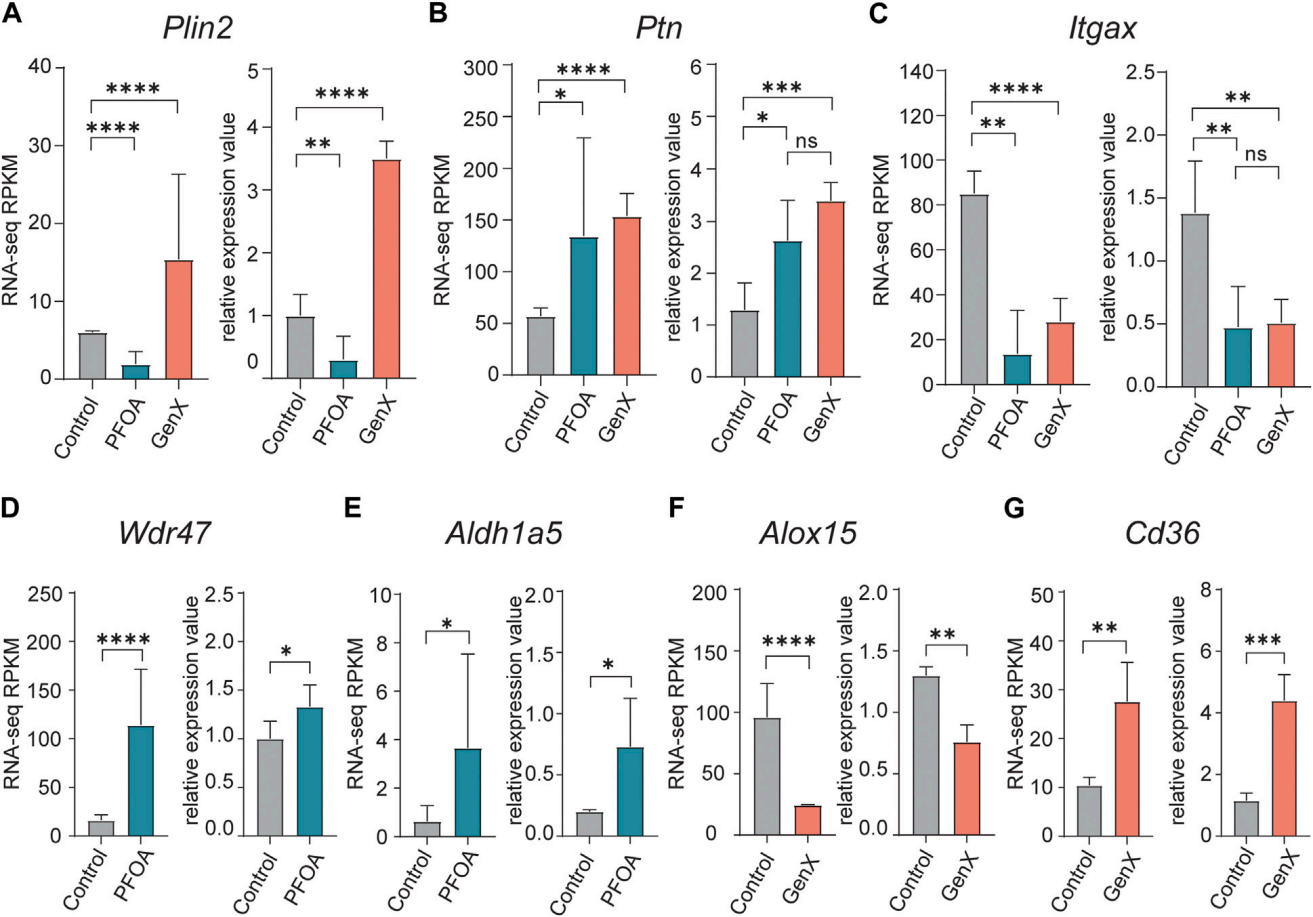
Quantitative reverse transcription PCR validation of upregulated and downregulated DEGs identified in the PFOA and GenX treatment groups. **(A–G)** Bar plots of qRT‐PCR relative quantification and RNA‐seq RPKM (Reads Per Kilobase of transcript per Million mapped reads) values for selected DEGs. **(A)** *Plin2*, **(B)** *Ptn*, **(C)** *Itgax*, **(D)** *Wdr47*, **(E)** *Aldh1a5*, **(F)** *Alox15*, **(G)** *Cd36*. For the RNA-seq RPKM level, significance was determined by *q*-values in edgeR (*, *q* < 0.05; **, *q* < 0.01; ****, *q* < 0.0001). For qRT-PCR validation experiments, significance was determined using an unpaired t-test (*p < 0.05; **, *p* < 0.01; ***, *p* < 0.001; ****, *p* < 0.0001).

At a cut-off of FDR <0.05 and log_2_(fold change) > 1, 578 DEGs, including 422 upregulated and 156 downregulated genes, were detected in the PFOA treatment group (Figures 2A,B; Supplementary Data S1). Using the same cut-off values as those used in the PFOA group, 148 DEGs, including 125 upregulated and 23 downregulated, were detected in the GenX treatment group (Figures 2C,D and Supplementary Data S2). Twenty-two genes were significantly upregulated, and 10 genes were significantly downregulated in both treatment groups (Figure 4A), suggesting that similar molecular mechanisms were used. Interestingly, two DEGs, *Plin2* and *Lgals9-like,* had opposing expression patterns (Figure 3B). While *Plin2* was downregulated by 4.9-fold after PFOA treatment, its expression nearly quadrupled after GenX treatment. *Lgals9-like* had a 4.5-fold reduction in expression after PFOA exposure, while GenX treatment doubled its expression. Overall, however, most overlapping DEGs had concordant expression patterns following PFOA and GenX treatment, with minor differences between the two groups.

### qRT-PCR validation of the DEGs

To confirm the DEGs identified in RNA-seq data, ten genes were selected for validation using qRT-PCR (see Methods). The opposing expression patterns of *Plin2* observed following treatment with PFOA and GenX was confirmed by qRT-PCR (*p* < 0.01, t-test; Figure 4A). Meanwhile, *Ptn* was upregulated and *Itgax* was downregulated in both the PFOA and GenX treatment groups (Figures 4B,C). The remaining genes selected for testing (*Wdr47*, *Aldh1a5*, *Alox15*, *Cd36*, *Cd38*, *Fabp5*, and *Itgb3*) were also confirmed by qRT-PCR (Figures 4D–G; Supplementary Figure S2). The validation results were consistent with the RNA-seq results.

### Biological pathways altered by PFOA/GenX treatment

**FIGURE 5:**
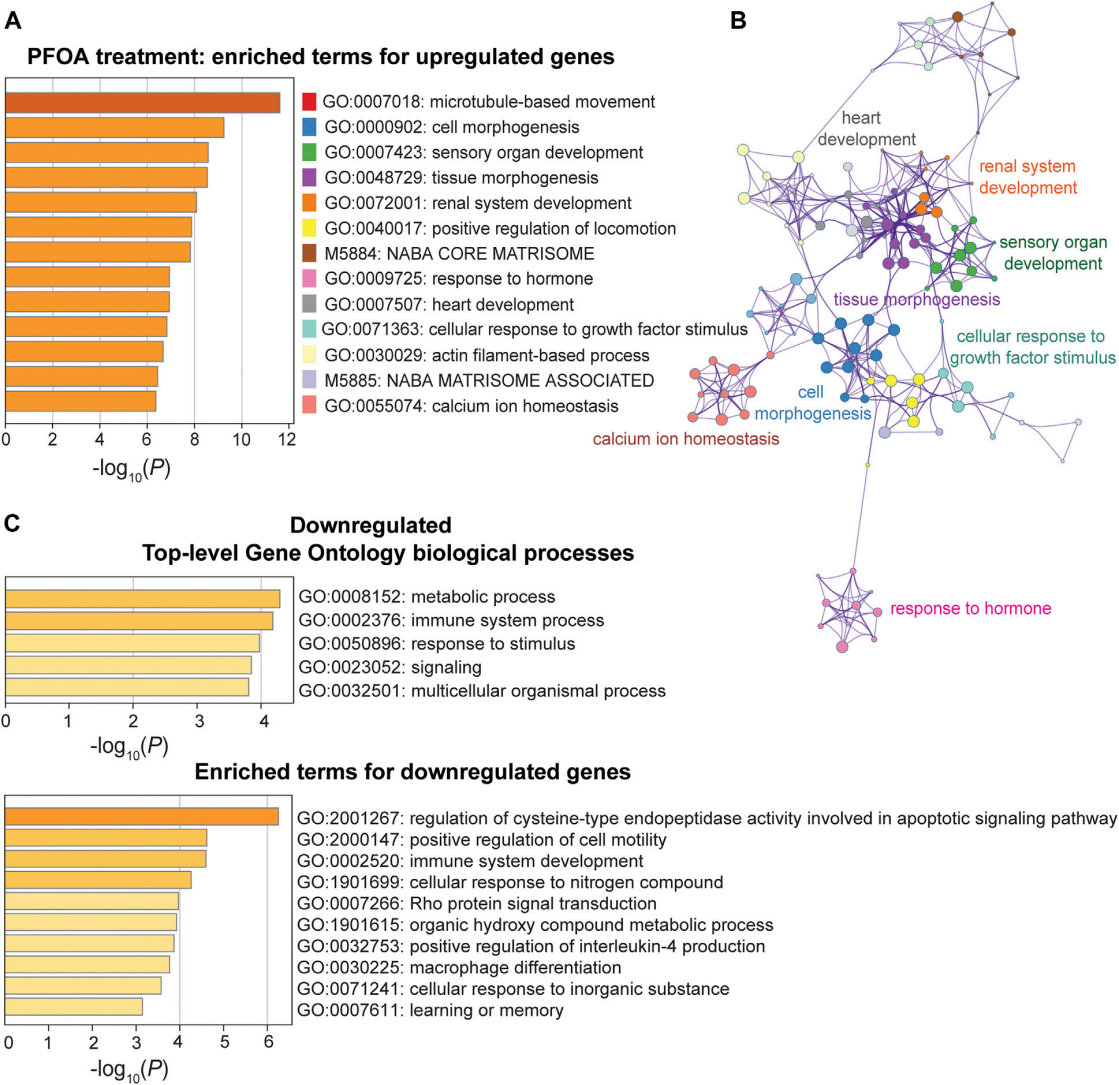
Pathway enrichment analysis of DEGs between the PFOA treatment and control groups. **(A, C)** Enriched functional categories for upregulated and downregulated genes in the PFOA treatment and control groups. Enrichment scores are measured by −log_10_(*p*-value). **(B)** The network plot of enriched terms for upregulated genes. GO terms were represented by the same color dots as in **(A)**.

In response to PFOA treatment, five of the top ten significantly upregulated GO terms (*p* < 10^−6^) were involved in developmental processes, including cell morphogenesis (GO: 0000902), tissue morphogenesis (GO: 0048729), sensory organ development (GO: 0007423), renal system development (GO: 0072001), and heart development (0007507) (Figure 5A). The GO interaction network illustrated that tissue morphogenesis was tightly associated with other developmental processes, such as other organ development and embryonic morphogenesis (Figure 5B), suggesting that development-related functional terms were affected. Microtubule-based movement (GO: 0007018) was the most significant GO term, and the actin filament-based process (GO: 0030029) was also enriched, indicating a potential upregulation of intracellular transport. Pathways that were downregulated after PFOA exposure included metabolic and immune system processes (*p* < 10^−4^; Figure 5C). The regulation of cysteine-type endopeptidase activity involved in apoptotic signaling (GO: 2001267) ranked highest among the downregulated GO terms (Figure 5C).

**FIGURE 6:**
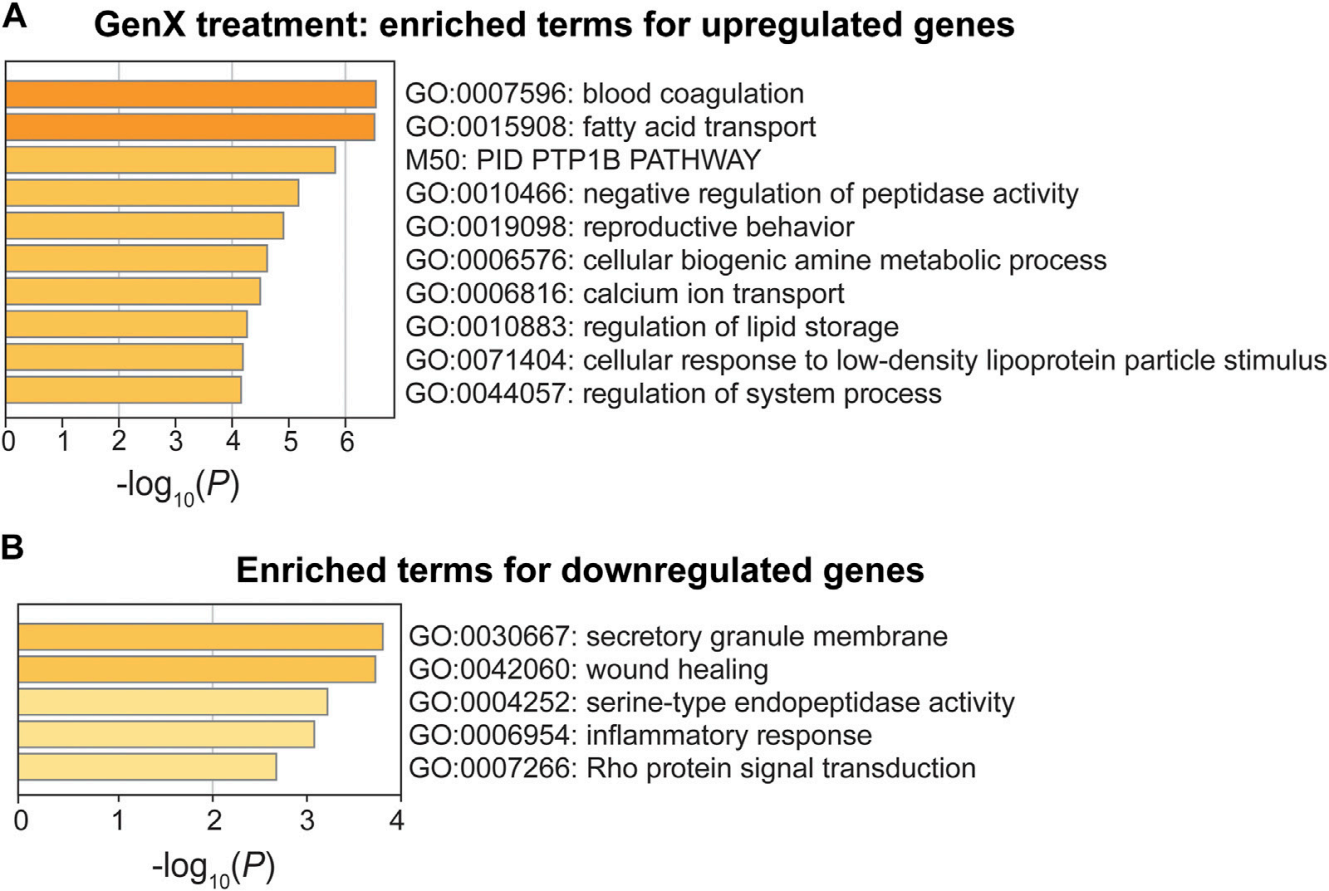
Pathway enrichment analysis of DEGs between the GenX treatment and control groups. **(A, B)** Enriched functional categories for upregulated genes and downregulated genes in the GenX treatment group compared to the control group. Enrichment scores are measured by −log_10_(*p*-value).

In the GenX treatment group, upregulated genes were enriched for GO terms involved in blood coagulation (GO:0007596), fatty acid transport (GO:0015908), and negative regulation of peptidase activity (GO:0010466) (*p* < 10^−5^; Figure 6A). One upregulated gene, *Cd36,* was involved in all three of the most significant pathways (Figure 4G). Three additional DEGs, *Fabp5*, *Fabp4*, and *Plin2*, were also involved in fatty acid transport. The DEGs, *Pros1*, *Cd9*, *Cd36*, and *Itgb3*, were involved in blood coagulation, a pathway that is closely linked to inflammation (Verhamme and Hoylaerts, 2009). Despite the limited number of downregulated DEGs in the GenX group (N = 22), the inflammatory response (GO:0006954), integrin-mediated signaling (GO:0007229), and wound healing (GO:0042060) pathways were significantly enriched (Figure 6B). Of the GenX downregulated genes, the lipoxygenases, *Alox15* and *Alox12,* in addition to *Lilrb4, Fn1*, and *Adra2a*, were involved in inflammatory response pathways. Integrin-mediated signaling genes, including integrin’s two subunits, *Itgax* and *Itgam*, were also downregulated.

## Discussion

### RNA-seq transcriptome analysis of PFAS exposure in a marsupial model

The laboratory opossum is a unique marsupial model for investigating the effects of environmental compounds given the availability of the reference genome and inbred laboratory strains, the ability to annotate genes, and the development of an established breeding protocol. To our knowledge, this is the first *in vitro* study that investigates the effect of PFAS chemicals on whole blood from the laboratory marsupial, *Monodelphis domestica*, providing important preliminary data for *in vivo* research. This study identified significant changes in the expression of hundreds of genes in whole blood culture following exposure to PFOA and GenX, serving as proof of principle for further *in vivo* toxicological studies.

### PFOA exposure altered the expression of genes involved in developmental processes

PFASs are present in both human serum and in the cord blood and milk of pregnant women (Monroy et al., 2008; Sundström et al., 2011), and can be transmitted from the mother to embryos across the placenta and to infants through breast milk (Gyllenhammar et al., 2018; Blake and Fenton, 2020). Serum PFAS levels in children generally exceed those of their mothers (Fromme et al., 2010). Prenatal exposure can affect fetal growth and may cause various adverse health issues, including a disruption in fetal hormone levels, depressed immune functionality, and increased susceptibility to liver injury (Pennings et al., 2016; Nian et al., 2020; Stratakis N et al., 2020), leading to a reduction in fetal growth and birth weight (Johnson et al., 2014; Verner et al., 2015; Li et al., 2017; Starling et al., 2017; Xu et al., 2019). Possible molecular mechanisms include altered maternal metabolic and hormonal profiles, as well as epigenetic modifications of developing embryos and fetuses (Kobayashi et al., 2017; Blake et al., 2020). The current study found that several genes associated with developmental processes were altered by PFOA exposure, including cell/tissue morphogenesis and organ/system development. However, it is important to note that gene expression in whole blood cultures is not a direct measurement of developmental processes. Further investigation of gene expression patterns at early embryonic development stages is required to assess the effect of PFOA on fetal development.

### Fatty acid transport functions are enriched in upregulated genes following GenX treatment

Lipid transport, metabolism, and liver function are some of the most susceptible targets to PFAS treatment (Guruge et al., 2006; Fragki et al., 2021). Human epidemiological studies have shown that PFOA and PFOS are positively associated with blood cholesterol levels (Nelson et al., 2010; Eriksen et al., 2013), and animal studies have identified impaired fatty acid homeostasis and transport in rodent models (Curran et al., 2008; Wan et al., 2012).

Previous studies in zebrafish embryos have confirmed that GenX and PFOA both cause a similar level of hepatotoxicity and lipid metabolism dysfunction (Gebreab et al., 2020). The upregulation of fatty acid transport and lipid storage pathways found in GenX-treated laboratory opossums is in accordance with previous findings. This study validated the major genes, including *Fabp5*, *Cd36*, and *Plin2*, that are regulated during fatty acid transport*.* CD36, or fatty acid translocase, is a membrane glycoprotein that helps to transport long-chain fatty acids through the adipocyte membrane and is the predominant protein responsible for facilitating fatty acid uptake (Hajri and Abumrad, 2002; Glatz and Luiken, 2018). Fatty acid binding proteins (FABPs) bind to CD26 to form a physical complex (Spitsberg et al., 1995), so they are often co-expressed and co-regulated in various tissues (Coburn et al., 2001). GenX treatment is associated with increased expression of both genes. Similar findings were reported in human and rodent liver cell lines (Wu et al., 2017; Sheng et al., 2018). Enhanced expression of CD36 and L-FABP were also observed in GenX-exposed chicken liver tissues, and this was abolished by peroxisome proliferator-activated receptor alpha (PPARα) silencing (Xu et al., 2021). PLIN2 (perilipins) is a ubiquitously expressed lipid droplet protein associated with lipid storage (Brasaemle et al., 1997). This protein protects lipid droplets from lipolysis in the liver, and its downregulation depletes hepatic triglyceride through enhanced autophagy in mouse MEF cells (Tsai et al., 2017). The current studies showed that *Plin2* was upregulated in response to GenX treatment, enriching the lipid storage pathway.

A potential limitation of this study is the use of DMSO to prepare the GenX treatment. While several studies have used DMSO as a solvent for GenX (Jabeen et al., 2020; Wen et al., 2020; Hvizdak et al., 2023), recent findings have shown that GenX (HFPO-DA) is degraded to Fluoroether E-1 within 2 h of exposure to >99.5% HPLC-grade DMSO (Liberatore et al., 2020). While GenX was newly prepared prior to use and was diluted immediately to 0.1% in cell culture media, GenX may not remain effective for the full 12-h treatment time. Thus, changes in gene expression observed in the blood transcriptomes may have occurred in response to the degradation product, Fluoroether E-1. EtOH or DI water may be more appropriate solvents for future GenX studies.

### Inflammatory responses and immune pathways are enriched in both PFOA and GenX treatment groups

PFAS, especially PFOA and PFOS, are associated with inflammation, cytokine expression, and adaptive and innate immune responses (Sunderland et al., 2019). There is mounting evidence demonstrating that PFAS have an immunotoxic effect on rodents, birds, and reptiles (DeWitt et al., 2009; DeWitt et al., 2012; Woodlief et al., 2021). PFOA and PFOS inhibit T-cell-dependent IgM antibody responses (TDAR) in mice, strongly indicating that immune function has been suppressed (DeWitt et al., 2009; Zheng et al., 2011). A thorough review of 25 epidemiological studies in humans illustrates that PFAS have an immunotoxic effect, reducing the production of antibodies and immune markers (Sunderland et al., 2019). A transcriptomic study in human cord blood also showed that prenatal exposure to PFAS can impair immune functionality in early childhood (Pennings et al., 2016).

The current study showed significantly decreased expression levels of *Lgals9*, *Zfp36l1, Cd40lg,* and other genes associated with immunity and inflammation following PFOA treatment. Galectin-9, encoded by *Lgals9*, belongs to the tandem-repeat subfamily of galectins and is highly expressed in the liver, where it has various effects on innate and adaptive immune responses (Golden-Mason and Rosen, 2017). This protein binds T cell immunoglobulin and mucin domain-containing molecule 3 (TIM-3), which are important immune regulators (Zhu et al., 2005). *Zfp36l1* helps to maintain the marginal-zone B cell compartment (Newman et al., 2017) and *Cd40lg* plays an important role in T cell-dependent immune responses and binds to CD40 on B cells (Miga et al., 2000; Zhou et al., 2009).

GenX treatment increased the expression of *Pros1*, *Cd9,* and *Cd36*, positive regulators of inflammation. Anticoagulant protein S (PROS1) negatively regulates the blood coagulation cascade (Castoldi and Hackeng, 2008) and serves as a ligand for tyrosine kinase (TAM) receptors, which play a fundamental role in regulating inflammatory responses (Lemke, 2013). *PROS1* is also upregulated in macrophages when inflammation resolves (Lumbroso et al., 2018). CD9 is a platelet antigen (Mangin et al., 2009), which associates with CD36 on the macrophage surface, leading to the formation of foam cells (Huang et al., 2011). The most significantly downregulated DEG in the inflammatory response pathway, *Alox15,* encodes a lipoxygenase*,* which acts to suppress inflammation (Tian et al., 2017). *Alox15* expression and metabolite levels were reduced by both PFOA and GenX in an *in vitro* study of human trophoblast migration (Szilagyi et al., 2020).

The current study found that the expression of several DEGs was similarly impacted by PFOA and GenX treatment, suggesting potential shared mechanisms. While downregulated genes, including *Scube2*, *Anpep*, and *Itgax*, showed more profound repression in response to PFOA than GenX, upregulated genes, including *Ttc12*, *Cdh4*, and *Fstl4*, were more significantly expressed in response to GenX treatment, suggesting that the two chemicals induced different levels of gene expression.

### Working toward an established *in vivo* model of fetal toxicology using *Monodelphis domestica*

This study broadened the spectrum of current research on the effects of PFOA and GenX treatment on gene expression in whole blood culture from the marsupial model, *Monodelphis domestica*. The findings that PFAS exposure can lead to significant alterations in developmental processes, fatty acids transport/storage, and immune and inflammatory pathways are consistent with previous rodent and human studies, reinforcing existing knowledge of the susceptible pathways and potential harm induced by PFAS exposure. *Monodelphis domestica* has several advantages over existing eutherian models in developmental toxicology research. It is an ideal model for evaluating the effects of environmental toxic compounds exposure on early embryonic development because the young are born in a very immature state on embryonic day 13.5, equivalent to a 5.5-week human embryo or an 11.5-day mouse fetus (Mate et al., 1994). Thus, this model provides a unique opportunity to access and examine early embryos without sacrificing the mother or disturbing the developing littermates. The lungs are not well developed when laboratory opossums are born, so much of the respiratory function occurs *via* the skin. Other major organs, including the brain and digestive and neuronal systems, are still developing (Saunders et al., 1989; Leo and Brunjes, 1999; Modepalli et al., 2018). The extra-uterine embryonic and fetal stages can facilitate time series studies using embryos/pups from the same litter. Another advantage is that laboratory opossums reach sexual maturity at 6 months of age, providing more resolution over laboratory mouse models. This *in vitro* study provided preliminary results that will inform future *in vivo* studies in developing embryos that focus on major organs (such as the lung, liver, brain, and kidney). This will help to elucidate the impact of gestational PFOA/GenX exposure *via* the mother on embryonic stages throughout early development.

## Data Availability

The datasets presented in this study can be found in online repositories. The names of the repository/repositories and accession number(s) can be found below: https://www.ncbi.nlm.nih.gov/, GSE208237.
