# Supplementary material for: Blood transcriptome responses to PFOA and GenX treatment in the marsupial biomedical model Monodelphis domestica
Source: Front Genet. 2023 Feb 15;14:1073461. doi: 10.3389/fgene.2023.1073461 (PMC9974665; doi:10.3389/fgene.2023.1073461)
Supplement: Supplementary file 1 [file DataSheet2.PDF]

Table S1. ***Monodelphis domestica*** **animals used in this study.**

Animal ID	Sex	Stage	Strain	Dam ID	Sire ID	Experiment
A0035	Male	adult	LSD	A0012	A0015	Experiment 1
A0036	Male	adult	LSD	A0012	A0015	Experiment 1
A0038	Male	adult	LSD	A0010	A0014	Experiment 1
A0039	Male	adult	LSD	A0010	A0014	Experiment 1
A0093	Male	adult	LSD	A0032	A0046	Experiment 2
A0094	Male	adult	LSD	A0032	A0046	Experiment 2
A0132	Female	adult	LSD	A0090	A0016	Experiment 2
A0135	Female	adult	LSD	A0015	A0089	Experiment 2
A0152	Female	adult	LSD	A0134	A0136	Experiment 2
A0153	Female	adult	LSD	A0134	A0136	Experiment 2

Table S2. The sample and RNA-seq library information.

Sample name	Experiment	Sex	Time Point	Dilution	Group	Treatment Conc.	Library Conc. (ng/ul)	Library Size	Mapped reads
A39TP0	Exp 1	Male	0h	1:2	-	-	57.2	304	66,726,740
A36TP0	Exp 1	Male	0h	1:2	-	-	90	217	306,787,960
A3538TP0	Exp 1	Male	0h	1:2	-	-	84.4	367	99,600,946
A39TP1	Exp 1	Male	12h	1:2	-	-	88	302	179,300,446
A36TP1	Exp 1	Male	12h	1:2	-	-	89.8	247	243,354,202
A3538TP1	Exp 1	Male	12h	1:2	-	-	87.6	308	377,251,968
A39TP2	Exp 1	Male	24h	1:2	-	-	65.2	314	135,652,668
A36TP2	Exp 1	Male	24h	1:2	-	-	90.8	309	176,557,434
A3538TP2	Exp 1	Male	24h	1:2	-	-	75.2	282	377,251,968
A39TP3	Exp 1	Male	48h	1:2	-	-	61.8	304	96,950,392
A36TP3	Exp 1	Male	48h	1:2	-	-	82.2	297	136,238,368
A3538TP3	Exp 1	Male	48h	1:2	-	-	91.2	395	119,747,544
A39TP4	Exp 1	Male	72h	1:2	-	-	78.8	241	141,064,840
A36TP4	Exp 1	Male	72h	1:2	-	-	79.8	479	109,639,450
A3538TP4	Exp 1	Male	72h	1:2	-	-	73.8	293	111,943,592
C_A9394R1_TP1	Exp 2	Male	12h	1:2	Control	-	6.2	376	27,739,880
C_A9394R2_TP1	Exp 2	Male	12h	1:2	Control	-	3.3	310	36,990,742
C_A9394R2_TP2	Exp 2	Male	24h	1:2	Control	-	3.2	296	19,898,378
C_A9394R3_TP2	Exp 2	Male	24h	1:2	Control	-	2.8	292	26,529,588
F_A9394R2_TP1_low	Exp 2	Male	12h	1:2	PFOA	400uM	2.6	286	14,994,876
F_A9394R2_TP1_high	Exp 2	Male	12h	1:2	PFOA	800uM	2.4	286	9,938,900
F_A9394R1_TP2_low	Exp 2	Male	24h	1:2	PFOA	400uM	2.6	306	6,078,826
F_A9394R1_TP2_high	Exp 2	Male	24h	1:2	PFOA	800uM	2.4	287	3,539,142
G_A9394R3_TP1_high	Exp 2	Male	12h	1:2	GenX	1200uM	4.3	283	19,956,274
G_A9394R4_TP1_high	Exp 2	Male	12h	1:2	GenX	1200uM	5.4	293	16,985,774

Table S3. The Quantitative Reverse Transcription PCR (qRT-PCR) primer sequences for the selected differentially expressed genes (DEGs).

Gene name	Primer sequences
PLIN2	F: CACTGAAGAAGAACTAGAAA<br>R: TGGCTTCATTAACCCTG
ITGAX	F: CTGCTTATCTGGGCTATTCCAT<br>R: GCTCCAAAATAGGAACCAACCTG
PTN	F: AAGAAAGAGAAACCAGAAA<br>R: TGGAACTGGTATTTGCACTC
ALDH1A5	F: AAGAAGAGATTTTTGGACCAG<br>R: CAAACAGTTCCAGCTTGCAG
WDR47	F: TTCCACCAACAAAGGCAT<br>R: ATAGCAGAAACCCAATACACC
CD36	F: CAGCTGCACCCATTCTC<br>R: TTGTAAACTCCATCTACCGTA
CD38	F: ACTTTGTAATAAGACTCTCC<br>R: GACTTTATCAGCCATGTAGCC
FABP5	F: ACATGAAGGAACTGGGA<br>R: TGCCATCAGCTGTGGTT
ITGB3	F: GAAGACAACCTGCTTACCCAT<br>R: CCAGCCAATTTTTTCATCACAG
ALOX15	F: GACCCAGTCGCGGAAAGGAA<br>R: TGATTCGCTCACAAAACCAGT

F: PCR forward primer, R: PCR reverse primer
