# Supplementary material for: Blood transcriptome responses to PFOA and GenX treatment in the marsupial biomedical model Monodelphis domestica
Source: Front Genet. 2023 Feb 15;14:1073461. doi: 10.3389/fgene.2023.1073461 (PMC9974665; doi:10.3389/fgene.2023.1073461)

Figure S1. Scatterplots of gene expression levels from RNA-seq data in Experiment 1.

(**A**) Comparisons within the same replicate at different time points 0h, 12h, 24h, 48h, and 72h.
(**B**) Comparisons between the two biological replicates (A0036 and A0039) at 12h, 24h, 48h, and 72h after culture. ρ: Spearman's rank correlation coefficient.

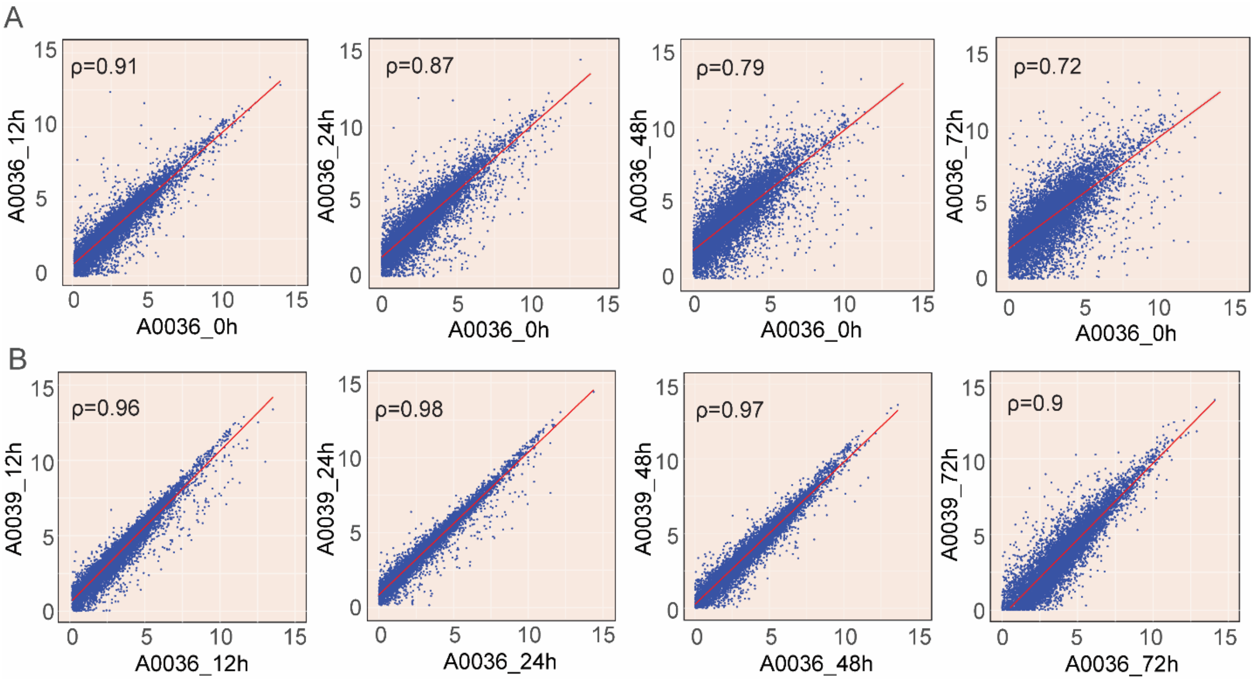

Figure S2. Barplot of qRT-PCR (Quantitative Reverse Transcription PCR) results for three differentially expressed genes (DEGs).

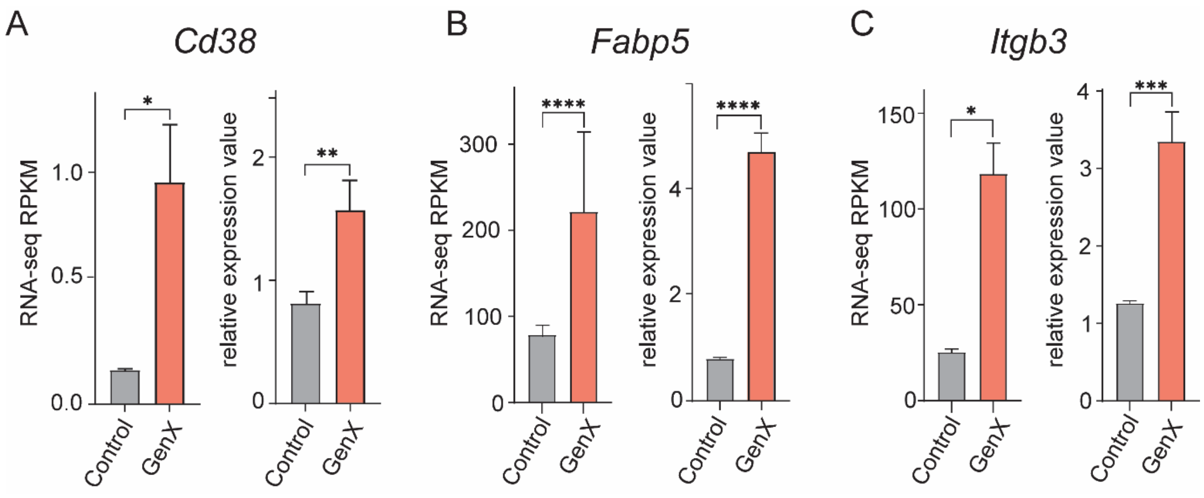

Supplement: Supplementary file 3 [file DataSheet1.PDF]
